# The global state of research in pain management of osteoarthritis (2000–2019): A 20-year visualized analysis

**DOI:** 10.1097/MD.0000000000023944

**Published:** 2021-01-15

**Authors:** Taoyu Chen, Jiaying Zhu, Yu Zhao, Haoqian Li, Pengcui Li, Jianjun Fan, Xiaochun Wei

**Affiliations:** a Department of Orthopedics, The first affiliated hospital of Baotou Medical College, Inner Mongolia University of Science and Technology, Baotou, Inner Mongolia; b Department of Orthopedics, Second Hospital of Shanxi Medical University; Shanxi Key Lab of Bone and Soft Tissue Injury Repair; c Department of Pharmacy; Second Hospital of Shanxi Medical University, Taiyuan, Shanxi Province, China.

**Keywords:** bibliometrics, global trends, osteoarthritis, pain management, visualized study

## Abstract

There has been a highly active area in the pain management of osteoarthritis (OA) over the past 2 decades. The study aims to unmask the global status and trends in this field.

Publications on pain management of OA from 2000 to 2019 were retrieved from the Web of Science (WOS) database. The data were analyzed using bibliometric statistical methodology. The software VOS viewer was used for bibliographic coupling, co-authorship, co-citation, co-occurrence analysis and to investigate the publication trends in pain management of OA.

A total of 8207 researches in amount were included. The relative research interests and number of publications indicated a rising trend. The USA made the greatest contribution to this field, with the most publications, total citations and the highest H-index, while Sweden had the highest average citation per publication. The most contributive organization was Boston University. The journal OA *and Cartilage* published the most relative articles. Researches could be grouped into 5 clusters based on co-occurrence network map: Health and Epidemiology; Sport Medicine; Clinical Study; Mechanism Research and Medical Technology and Science. Medical Technology and Science was predicted as the next research topic in this field.

The number of publications about pain management of OA would be increasing based on current global trends. The USA made the largest contribution to this field. The development of Medical Technology and Science may be the next popular topics in the pain management of OA research.

## 1 Introduction

Osteoarthritis (OA) is an abnormal remodeling of joint tissue within the affected joint including pathologic changes such as degradation of the articular cartilage, thickening of the subchondral bone, formation of osteophytes, variable degrees of inflammation of the synovium, degradation of ligaments and hypertrophy of the joint capsule.^[1]^ Knees, hips, spines and joints in the hands are the commonly affected anatomic sites. Knee OA is the most common joint disorder in elderly individuals, and overall prevalence of symptomatic knee OA is 8.1% based on China Health and Retirement Longitudinal Study (CHARLS) among Chinese adults.^[2,3]^ Pain is a distressing experience association with actual or potential tissue damage with sensory, emotional, cognitive and social components,^[4]^ which is the first and predominant symptom of OA that causes patients to seeking medical care^[5]^ and broadly contributes to the global burden of musculoskeletal conditions.^[5,6]^ Pain in OA comes from several sources, both peripheral and central.^[7]^ Pain can affect the quality of life of patients seriously, which indirectly increases the occurrence rate of cardiovascular event and all-cause mortality.^[8–10]^

The main purpose of knee OA pain management is to alleviate joint pain, improve daily function and quality of life by increasing muscle strength, physical activity and emotional functioning.^[11]^ Knee OA pain relief often involves a mix of^[12]^ surgical treatment (joint replacement, arthroscopic surgery and osteotomy^[13–15]^); pharmacological treatment (NSAIDs and opioid analgesics^[16,17]^); and nonpharmacological treatment (physical therapy and self-management education^[18–20]^). However, the global development trend regarding pain management for OA has not been well studied yet. Thus, it is needed to summarize the current status of pain management in OA and to predict promising keywords and trends.

Bibliometric analysis can provide information based on literature database and literature metrology characteristics, which is used to qualitatively and quantitatively estimate trends in recent years research activity. It provides a way to seize development in a certain field and to compare the contributions of scholars, journals, institutions, and countries. In recent years, bibliometric analysis has been successfully applied in several research areas to assist the development of clinical policies and guidelines, and also been used in evaluating research trends in electronic health,^[21]^ bio-receptor,^[22]^ microbiome-gut-brain axis^[23]^ and stem cell.^[24]^ Therefore, we conduct a brief discussion of pain management research in knee OA, unmasking trends that could be helpful for gaining a wide knowledge of global developments in the field and future directions.

## 2 Methods

### 2.1 Data source and search strategy

Bibliometric analysis was conducted using the Web of Science (WOS) Core Collection, which is considered as the optimal database for bibliometric analysis.^[25]^ All publications were searched in the WOS from 2000 to 2019, including the researches in this field in recent 20 years. In this study, the retrieval types were as follows: (TS = knee OA OR KOA) and (TS = pain OR pain treatment OR pain management OR pain assessment) and (Language = English) and (Document types = Article) and (publishing year = 2000–2019). Country-specific information was also retrieved through the WOS index.

### 2.2 Data collection

The data mining of all included publications was downloaded from the WOS database such as title, author, year of publication, nationalities, affiliation, journal, keywords and abstract. Graph Pad Prism 5 and Origin Pro 2018 were used to analyze the data after manually cleaned. Ethical approval was not required as no human and animal subjects were enrolled.

### 2.3 Bibliometric analysis

The basic features of publications were described by using the intrinsic function of WOS. The H-index reflects both the number and citation impact of publications.^[26,27]^ Relative research interests was calculated as the number of publications in a certain field divided by all-field publications per year. Use Microsoft Excel (Office2019) to build a forecasting trend model and plot the time curve of the publication: *Y* = 55.789*X* − 93.611, *R*^2^ = 0.9996. In this formula, the independent variable *X* represents the year and *Y* represents the number of publications per year.

### 2.4 Visualized analysis

VOS viewer software 1.6.15 (Leiden University, Leiden, The Netherlands) was used for bibliometric visualization and analysis of the literature.^[28]^ In this research, VOS viewer was used for co-citation, co-authorship, bibliographic coupling and co-occurrence analysis.^[29]^

## 3 Results

### 3.1 Trend of global publication

#### 3.1.1 Global publications by country

**Figure 1:**
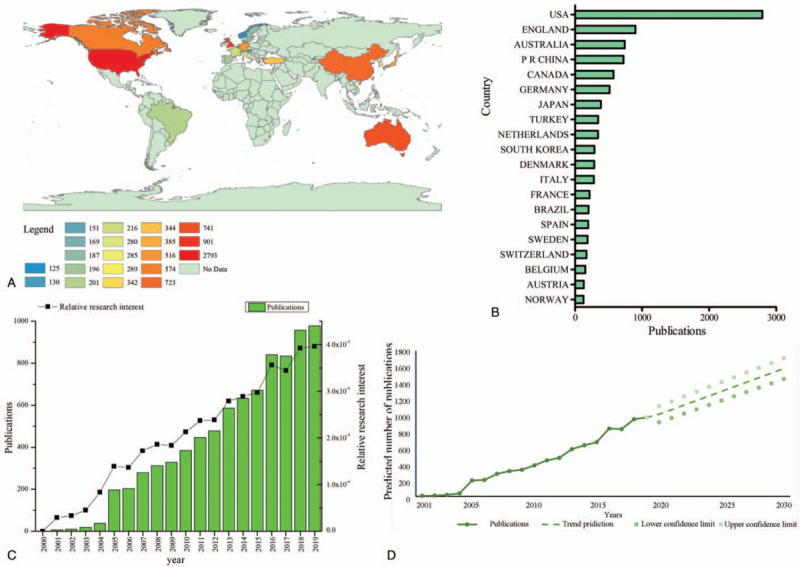
Global trends and countries contributing to osteoarthritis (OA) and pain management research. (A) World map showing the distribution of OA and pain management research. (B) The sum of OA and pain management research-related articles from the top 20 countries. (C) The global number and relative research interests of publications related to OA and pain management research. The green bars indicate the single-year publication numbers, and the black curve indicates the relative research interests. (D) A model-fitting curve of global publication growth trends to predict publication numbers in future.

Sixty-seven countries and regions in amount published articles in this field. Among these countries, the USA published the largest number of articles (2793, 34.03%), followed by England (901, 10.98%), Australia (741, 9.03%), China (723, 8.81%), and Canada (574, 6.99%) (Fig. 1A,B).

#### 3.1.2 Global publications by year

A total of 8207 articles from 2000 to 2019 in amount met the search criteria. From 2000 to 2019, a remarkable increasing trend of global publications per year was found. The number of publications increased from 0 (2000) to 978 (2019). Moreover, the relative research interests of this field were increasing during last few years (Fig. 1C).

#### 3.1.3 Global trends of publications

The time curve of the number of the publications was created by Microsoft Excel, which could help predict the future trend. Figure 1D showed the model fitting curves of the growth trend. Based on the time curve, the number of publications in this field was estimated to grow steadily.

### 3.2 Analysis of global publication

#### 3.2.1 Institution

**Figure 2:**
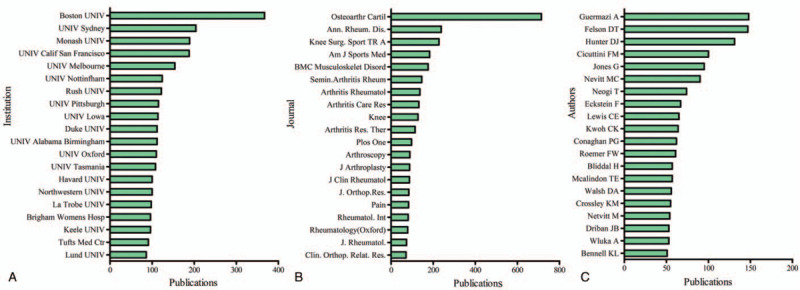
Publication amount of different institutions, journals, and authors. (A) The sum of OA and pain management research-related articles from the top 20 institutions. (B) The sum of OA and pain management research-related articles from the top 20 journals. (C) The sum of OA and pain management research-related articles from the top 20 authors.

**Figure 3:**
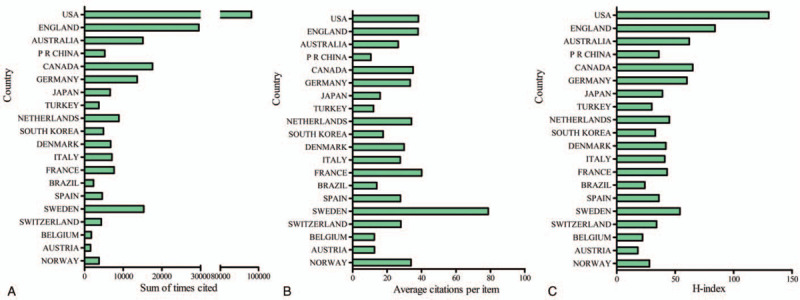
Citation frequency and H-index levels of different countries. (A) The sum times cited of OA and pain management research-related articles from the top 20 countries. (B) The average citations per item for OA and pain management research articles from different countries. (C) The H-index of publications in the different countries.

The top 20 contributive institutions are listed in Figure 3C. The Boston University published the most papers (376 papers), and the University Sydney ranked second (204 papers), while Monash University ranked third (189 papers), followed by University Calf San Francisco (188 papers) and University Melbourne (154 papers) (Fig. 2B).

#### 3.2.2 Journal

OA and Cartilage published the most studies with 714 publications. There were 238 articles in Annals of The Rheumatic Disease, 227 articles in Knee Surgery Sports Traumatology Arthroscopy, 183 articles in American Journal of Sports Medicine, 176 articles in BMC Musculoskeletal Disorders on OA and pain management research. The top 20 journals with most studies are listed in Fig. 2C.

#### 3.2.3 Authors

A total of 1485 publications were from the top 20 authors, accounting for 18.09% of all publications in this field (Fig. 3D). Three authors who published the most research were Guermazi A with 148 publications, followed by Felson DT with 147 publications and Hunter DJ with 131 publications (Fig. 2D).

### 3.3 Quality of publications by country

#### 3.3.1 Total citation frequency

Publications from the USA had the highest number of citations (96,303), while England ranked second (29,585), followed by Canada (17,578), Sweden (15,345), and Australia (15,103) (Fig. 3A).

#### 3.3.2 Average citation frequency

Publications from Sweden (78.69) had the most top average number of citations. France (40.1) ranked second, followed by the USA (38.15), England (37.98), and Canada (35.01) (Fig. 3B).

#### 3.3.3 H-Index

The relevant publications from the USA (130) had the most top number of H-index, followed by England (84), Canada (65), Australia (62), and Germany (60) (Fig. 3C).

#### 3.3.4 Bibliographic coupling analysis

Bibliographic coupling is an arrangement that uses citation analysis to establish a “coupled” relationship between documents indicating these 2 publications own a common theme. The publication strength link indicates the number of its cited references in common with another publication. Vos viewer was applied to analyze the link strength of journals, institutions, or countries which have published researches in a common field reflecting the bibliographic coupling degree.

#### 3.3.5 Institution

**Figure 4:**
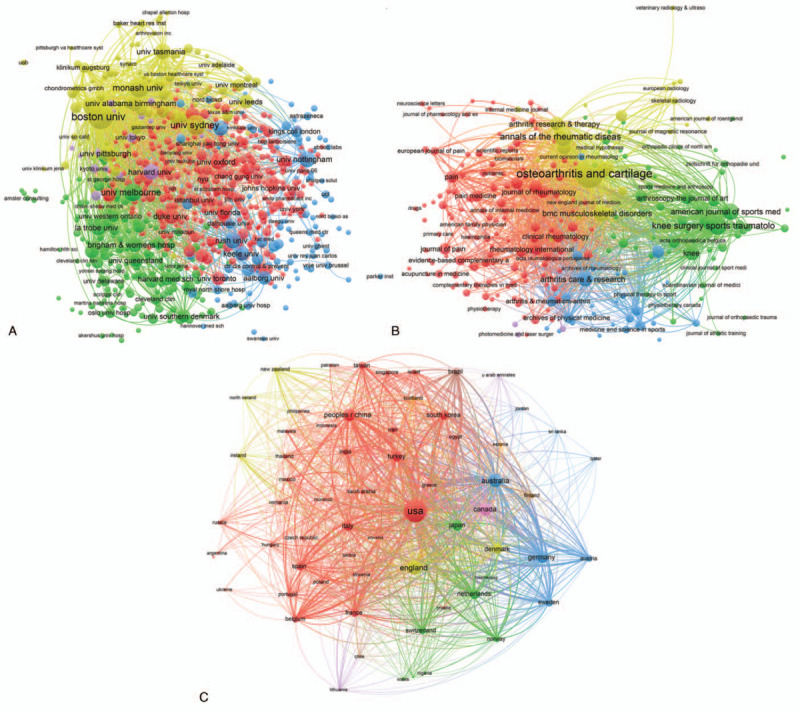
Bibliographic coupling analysis of global research about OA and pain management research. (A) Mapping of the 720 identified institutions on OA and pain management. (B) Mapping of the 255 identified journals on OA and pain management. (C) Mapping of the 64 countries on OA and pain management. The line between two journals/institutions/countries had established a similarity relationship. The thicker the line, the closer the link between the two journals/institutions/countries.

Total link strength was shown for 720 institutions, with each selected institution owing at least 5 papers in this field. The top 5 institutions with the most total link strength were the following: Boston University (940912 times); University Sydney (523023 times); Monash University (509989 times); University Calf San Francisco (492158 times); University Melbourne (404647 times) (Fig. 4A).

#### 3.3.6 Journal

Total link strength was shown for 255 journals, with each selected journal owing at least 5 papers in this field. The top 5 journals with large total link strength. The top 5 journals with large total link strength were the following: OA *and Cartilage* (299,788 times)*; BMC Musculoskeletal Disorders* (126,861 times)*; Arthritis Care and Research* (118,119 times)*; Knee Surgery Sports Traumatology Arthroscopy* (113,200 times)*; American Journal of Sports Medicine* (87,850 times). (Fig. 4b)

#### 3.3.7 Country

Total link strength was shown for 64 countries, with each selected country owing at least 5 papers in this field. The top 5 countries with large total link strength were the following: the USA (1817853 times); Australia (777616 times); England (682022 times); Canada (525319 times); China (500276 times) (Fig. 4C).

#### 3.3.8 Co-authorship analysis

Co-authorship analysis manifests the publication links between authors based on their number of co-authored papers. Vos viewer was applied to analyze the link strength of authors, institutions, or countries.

#### 3.3.9 Author

**Figure 5:**
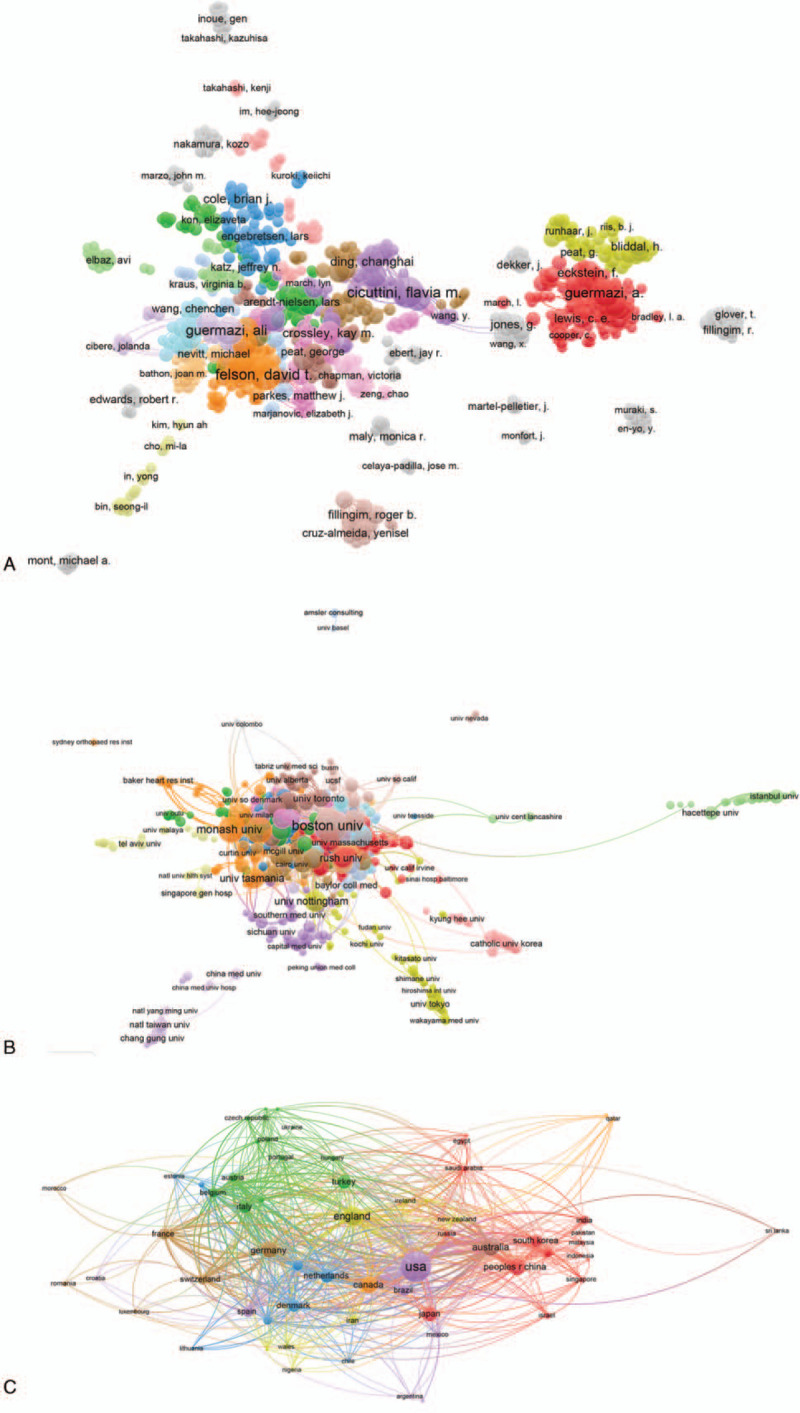
Co-authorship analysis of global research about OA and pain management research. (A) Mapping of the 935 authors co-authorship analysis on OA and pain management research. (B) Mapping of the 720 institutions co-authorship analysis on OA and pain management research. (C) Mapping of the 64 countries co-authorship analysis on OA and pain management research. The size of the points represents that two authors/institutions/countries had established collaboration. The thicker the line, the closer the link between the two authors/institutions/countries.

Total link strength was shown for 935 authors, with each selected author owing at least 5 papers included in this field. The top 5 authors with largest total link strength were the following: Cicuttini FM (359 times); Felson DT (355 times); Guermazi A (339 times); Nevitt MC (310 times); Hunter DJ (270 times) (Fig. 5A).

#### 3.3.10 Institution

Total link strength was shown for 720 institutions, with each selected institution owing at least 5 papers included in this field. The top 5 institutions with large total link strength were the following: Boston University (1154 times); University Calf San Francisco (631 times); University Sydney (596 times); Monash University (438 times); University Iowa (383 times) (Fig. 5B).

#### 3.3.11 Country

Total link strength was shown for 64 countries, with each selected country owing at least 5 papers included in this field. The top 5 countries with large total link strength were the following: the USA (1361 times); England (762 times); Australia (692 times); Germany (539 times); Canada (463 times) (Fig. 5C).

#### 3.3.12 Co-citation analysis

Co-citation analysis indicates that the link of items based on number of times they were cited in 1 document. Vos viewer was applied to analyze the total co-citation link strength of references or journals.

#### 3.3.13 Reference

**Figure 6:**
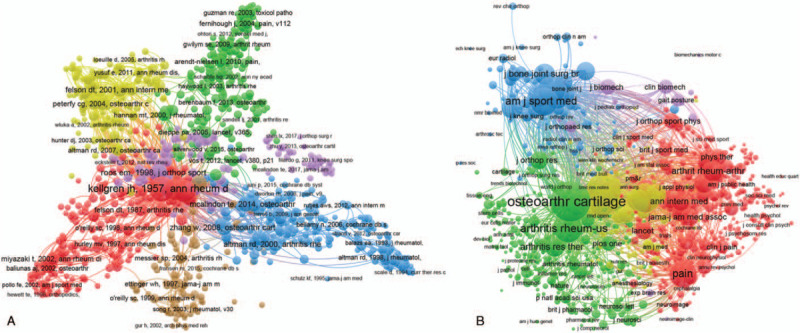
Co-citation analysis of global research about OA and pain management research. (A) Mapping of the 1372 publications by co-authorship analysis. A line between two icons indicates that both were cited in one paper. (B) Mapping of the 1165 identified journals by co-citation analysis. A line between two icons indicates that both were cited in one journal. The size of the icon indicates the citation frequency.

Total link strength was shown for 1372 references, with each selected reference owing at least 20 papers co-cited in this field.^[30]^ The top 5 studies with the largest total link strength were the following: Kellgren JH, 1957, ann rheum dis, v16, p494,^[31]^ (1020 times); Altman R, 1986, arthritis rheum, v29, p1039,^[32]^ (779times); Bellamy N, 1988, J rheumatol, v15, p1833,^[33]^ (734 times); Zhang W, 2008, osteoarthr cartilage, v16, p137,^[34]^ (298 times); Felson DT, 2001, ann intern med, v134, p541,^[35]^ (259 times) (Fig. 6A).

#### 3.3.14 Journal

Total link strength was shown for 1165 journals, with each selected journal owing at least 20 co-citations in this field. The top 5 journals with large total link strength were following: OA *and Cartilage* (735,096 times); *Annals of The Rheumatic Diseases* (489,351 times); *Arthritis Rheum-Us* (354,700 times); *Journal of Rheumatology* (345,487 times); *Pain* (324,649 times) (Fig. 6B).

### 3.4 Co-occurrence analysis

**Figure 7:**
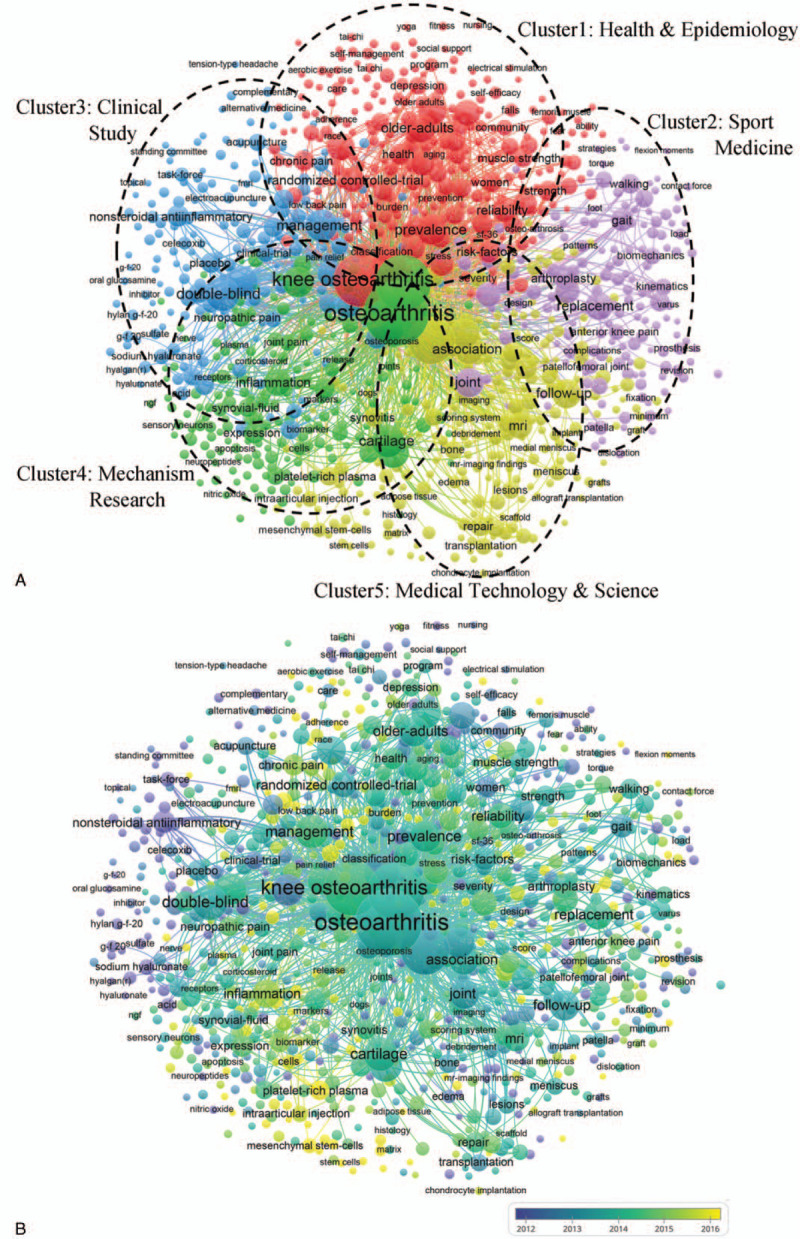
Co-occurrence analysis of global research about OA and pain management research. (A) Mapping of keywords in the research about OA and pain management; the size of the points represents the frequency, and the keywords are divided into 5 clusters: health care & nursing (upper in red), sport science (right in purple), clinical study (left in blue), mechanism research (left in green) and medical technology & science (lower in yellow). (B) Distribution of keywords according to the mean frequency of appearance; keywords in blue appeared earlier than those in yellow.

Co-occurrence analysis shows that the relationship of items based on the number of publications in which they occur together.^[36,37]^ Keywords were analyzed using Vos viewer, which were set as minimum occurrences number was 5 in total publications. As shown in Figure 7A, total 2133 identified keywords were classified into 5 clusters, approximately: “Health and Epidemiology”; “Sport Medicine”; “Clinical Study”; “Mechanism Research”; and “Medical Technology and Science.” In the “Health care and Nursing” cluster, the most used keywords were older-adults, health, prevalence. In the “Health and Epidemiology” cluster, the most used keywords were older-adults, health, prevalence, and woman. In the “Sport Medicine” cluster, the most used keywords were replacement, gait, walking, and kinematics. In the “Clinical Study” cluster, the most used keywords were double-blind, management, and clinical trial. In the “Mechanism Research” cluster, the most used keywords were knee OA, cartilage, inflammation. In the “Medical Technology and Science” cluster, the most used keywords were MRI, imaging, transplantation, association. These results showed the research field distribution of publications related to pain management of knee OA.

Keywords in total included publications were marked specific colors based on average appearing time (Fig. 7B). The blue color means the keyword appeared early and yellow-colored keywords appeared later. Before 2012, namely in the early stage of research, most studies focused on “Clinical Study.” The latest trends showed that the “Mechanism Research” and “Medical Technology and Science” clusters would be concerned widely in the future.

## 4 Discussion

### 4.1 Trends in pain management for OA

Bibliometric and visualized analysis can unmask the current status and predict hot topics in research field. With population aging, studies in OA pain may bring clinical guidelines. Thus, our study was conducted to evaluate pain management studies in OA about contributing countries, institutions, authors, journals, and research focus. As shown in our study, a significantly increasing number of publications per year is presented. In addition, the relative research interests was increasing dramatically recent years, especially in 2004 to 2018. A total of 64 countries had published relative studies in this field. Based on the current data, more studies with in-depth pain management knowledge in OA will be published in the coming year. The present optimistic results in turn will also allow investigators to conduct further high-quality research.

### 4.2 Quality and status of global publications

The total number of citations, average citations per paper, and H-index of a country represent its academic impact and quality of publications. The USA made the greatest contributions to the global research in terms of total number of publications as well as total citation frequency and H-index. Well, Sweden has the highest average number of citations. Thus, the USA can be regarded as the leading country in this field, and Sweden also played a significant role owning to high average citation frequencies. China ranked fourth in total number of publications. However, the total citation, h-index, and average number of citations ranked twelfth, twelfth, and twentieth, respectively. The discrepancy between the quantity and quality of publications may be due to an important reason. The academic evaluation system in China has focused on publish quantity of publications instead of quality.^[38]^

The OA and Cartilage, Annals of The Rheumatic Disease, Knee Surgery Sports Traumatology Arthroscopy, American Journal of Sports Medicine, and BMC Musculoskeletal Disorders published more studies on pain management of OA. The number of papers in OA and Cartilage is as many as the sum number of another four journals. The journals in Figure 2C may be main channels of publication for future researches in this field. Future studies in this field are more likely to be reported by the list journals.

Almost all the top 20 institutions including 18 universities and 2 medical centers were from the USA, England, and Australia, except Lund University in Sweden, which implying that establishing top class research institutions is essential to improving a country's academic level. Guermazi A, Felson DT, and Hunter DJ are the top 3 authors who published the most articles in this field. The top 20 authors could be regarded as the pioneers in pain management of OA. Their future studies may have a substantial impact on the development in this field and should be closely monitored to grasp the latest advancement.

In the present study, the relatedness between papers with regards to country, institution, and journal was established by bibliographic coupling analysis. Bibliographic coupling analysis constructed on the shared references among publications, providing deeper insights on how authors use and build links among the existing literature. The analysis showed that OA *and Cartilage* published most relevant articles, and the USA was in the leading position in this field. Co-authorship analysis was used to assess collaboration among countries, institutions, and authors. The one with greater total link strength suggested that the country/institution/author would be more likely to cooperate with others. Co-citation analysis was conducted to evaluate the academic influence of studies. The top studies with large co-citation frequency could be regarded as the landmark studies about pain management of OA. OA *and Cartilage* was the journal with the highest citation frequency in this domain.

### 4.3 Research focus on OA and pain management

Based on the co-occurrence analysis, we discovered directions and hot topics in this field. The keywords in title and abstract in all included studies were analyzed to create a map of a co-occurrence network. As the co-occurrence map shown, 5 research directions were observed, including “Health and Epidemiology”; “Sport Medicine”; “Clinical Study”; “Mechanism Research,” and “Medical Technology and Science”. The present results could help clarity the trend of future research. In the center of the co-occurrence map, the keywords, including knee OA, pain, arthritis, double-blind, and cartilage, were shown more prominently with higher weight. Thus, further high-quality studies evaluating pain management for OA in these 5 directions are still required.

The overlay visualization map was identical to the co-occurrence map except that items were colored differently. This method was important for monitoring the progress of research. In this overlay visualization shown in Figure 7B, colors indicate publication years. According to the results, “Medical Technology and Science” (yellow color) may be the next popular topic in this field. The “Medical Technology and Science” studies involving pain management in OA have been emerging widely, especially the technology development of intra-articular injection, stem cells, and platelet-rich plasma.^[39–41]^ In addition, technology-based on regenerative medicine and transforming medicine such as biomimetic tissue engineering is required. “Mechanism Research” plays a key role in pain management of knee OA. Primary studies investigating the pain mechanism and relation between mechanism and symptoms remain the focus of this research filed.^[2,42–45]^

Based on the finding of our study, the increasing number of publications indicated that the pain management serves as a key position in knee OA research. The positive finding in turn encourages investigators to perform further studies. The bibliometric and visualized analyses could provide the researchers with the knowledge of leading countries, famous authors, and top institutions in the field. With this knowledge, novice researchers can have a visual picture and gain insight more quickly. In addition, the co-occurrence analysis and overlay visualization map identify the prevalent trend and future research direction, which is helpful for the funding sources to make reasonable investment plan and provide support for formulation of pain management policy.

### 4.4 Strengths and limitations

Although the present study evaluated the status and trends of studies about pain management in OA via bibliometric and visualized analyses, the following items about limitations have to be mentioned. English language studies were included based on the database of WOS. Non-English language literature could have been omitted, leading to language bias. Additionally, differences may exist between the real world and the present results. Therefore, we still need to focus on the latest primary studies and other non-English studies in our daily research work.

## 5 Conclusion

The present study unmasked the global trends in pain management for OA. The USA was the largest contributor to studies and had the leading position in global research in this field. The journal OA *and Cartilage* had the largest number of publications related to this topic. We can believe that more studies about pain management for OA will be published in the coming years. Particularly, the “Medical Technology and Science” studies, involving pain management for OA is the next popular hot spot.

## Author contributions

**Conceptualization:** Taoyu Chen, Jiaying Zhu.

**Data curation:** Jiaying Zhu, Jianjun Fan.

**Formal analysis:** Taoyu Chen.

**Funding acquisition:** Pengcui Li.

**Investigation:** Yu Zhao.

**Methodology:** Taoyu Chen.

**Project administration:** Pengcui Li, Xiaochun Wei, Jianjun Fan.

**Resources:** Yu Zhao.

**Software:** Haoqian Li.

**Supervision:** Pengcui Li, Xiaochun Wei.

**Validation:** Haoqian Li, Xiaochun Wei.

**Visualization:** Taoyu Chen, Haoqian Li.

**Writing – original draft:** Taoyu Chen, Jiaying Zhu, Xiaochun Wei.

## Abbreviations

Abbreviations: OA = osteoarthritis, WOS = Web of Science.
